# 警惕具有进展为髓系肿瘤倾向的基因改变：骨髓增生异常综合征合并GATA2缺陷综合征一例

**DOI:** 10.3760/cma.j.issn.0253-2727.2021.12.015

**Published:** 2021-12

**Authors:** 浛 姚, 曦 杨, 曦 张, 平 王, 晓娟 邓, 梦林 罗, 婷 陈, 雨青 刘, 一梅 冯, 蕾 高, 佩艳 孔

**Affiliations:** 1 陆军军医大学附属新桥医院血液病医学中心，重庆 400037 Hematology Medical Center of Army Medical University Affiliated Second Hospital, Chongqing 400037, China; 2 重庆医科大学附属儿童医院风湿免疫科 400015 Department of Rheumatology and Immunology, Chongqing Medical University Affiliated Children's Hospital, Chongqing 400015, China; 3 陆军军医大学第二附属医院药学部，重庆 400037 Department of Pharmacy, Army Medical University Affiliated Second Hospital, Chongqing 400037, China

## 病例资料

患者为16岁男性，幼时罹患结核；胞弟11岁死于重症水痘，父亲有口腔溃疡发作史，祖母在29岁死于感染。2020年3月在当地医院确诊为骨髓增生异常综合征-难治性血细胞减少伴多系病态造血（MDS-RCMD），WPSS高危（3分）、IPSS-R中危（3.5分），伴7q−、+8阳性。行1个疗程地西他滨25 mg·m^−2^·d^−1^×5 d治疗，骨髓抑制期度过后疗效评估为部分血液学恢复的完全缓解（CRh）。血象虽恢复，但患者反复高热。2020年5月为行异基因造血干细胞移植至我院就诊。多次血液微生物送检阴性，结核菌素试验、结核干扰素结合试验阴性；免疫指标为轻度异常：抗核抗体谱Ro-52+、抗线粒体M2亚型抗体（AMA-M2）+，抗核抗体滴度（ANA-PD-S）1∶100，抗核抗体核型（ANA-PH-S）粗颗粒型，抗中性粒细胞抗体、甲状腺功能5项阴性。期间先后接受亚胺培南西司他丁、伏立康唑、替考拉宁（对万古霉素过敏）治疗，经多学科会诊更换为伊曲康唑、头孢哌酮舒巴坦、莫西沙星抗感染治疗，覆盖罕见菌；考虑不除外自身免疫异常所致发热，加用甲泼尼龙0.5 mg·m^−2^·d^−1^治疗。患者体温恢复正常，一般情况可。同步行异基因造血干细胞移植准备，于骨髓库寻找到一全相合女性供者，体检合格。

2020年8月患者再次因反复发热伴肛周脓肿就诊于我院，入院行利奈唑胺联合头孢哌酮舒巴坦抗感染后体温控制，但肛周脓肿反复，并形成多个浅表性瘘管。于普外科先后行脓肿抽液以及肛周脓肿切开术，但切开术后患处出现伤口持续渗液、延迟愈合（切开术后半月余）。

2020年11月因“高热4 d、皮肤软组织感染”第3次入院。查见肛周脓肿切开处有渗液以及次新伤口；耻骨联合上方见一皮温增高、中心疖肿、周围软组织红肿硬块（总直径约4 cm）。以美罗培南、利奈唑胺联合伊曲康唑抗感染效果不佳，将美罗培南换为替加环素，体温控制3 d后再次出现高热，复查肛管MRI提示复杂肛瘘形成。行肛瘘切开引流术，伊曲康唑换为伏立康唑，美罗培南每8 h 2 g微泵输注，替加环素换为多黏菌素，伏立康唑换为米卡芬净，后头孢他啶阿维巴坦替代美罗培南。但经上述调整后，患者发热频次、体温高峰无明显变化，且患者出现多处皮肤软组织感染，先后顺序为肛周、耻骨联合上、左侧乳房、左侧脸颊、右大腿外侧、右大腿内侧、唇周，除肛周病灶外，其余病灶发生过程多以表面少许疖肿，后周围组织出现红肿及硬块，随之化脓并形成破溃为共同表现。肛瘘手术伤口处见直径4～6 cm创面，深度3 cm，伴少许分泌物以及偶有渗血；伴随高热、全身多发软组织脓肿的同时，患者血象呈进行性下降，流式淋巴细胞亚群检测提示B细胞以及单核细胞群明显减少。

由于患者原发病诊断为MDS，在地西他滨治疗后再无化疗或免疫抑制治疗的前提下，反复高热、自发性多发软组织感染以及对广谱抗生素治疗无效等临床特征，考虑患者存在免疫缺陷可能。我们做了以下工作：（1）软组织感染处病灶活检排查微生物感染，结果提示为化脓性改变，组织培养未检出微生物；（2）送检患者免疫缺陷基因NGS检测，结果提示该患者存在多发基因改变，加送口腔黏膜检测，发现GATA2和EPG5为胚系来源（图1、图2）；（3）针对该患者存在的突变，加做患者本人口腔黏膜检测发现其中GATA2和EPG5为胚系突变；对患者父母进行相关基因突变验证，结果发现其父亲存在GATA2同样位点缺失，母亲存在EPG5同样位点缺失（图2）。（4）为患者提供以下治疗方案选择：①脐血输注；②间充质干细胞输注；③小剂量供者造血干细胞输注。有鉴于患者经济原因以及现有条件考虑，选择为分次小剂量输注供者造血干细胞输注。因此，在2020年12月31日回输1份冻存干细胞，其中含单个核细胞（MNC）0.693×10^8^/kg，CD34^+^细胞0.49×10^6^/kg，此后于1月8日、1月22日、2月23日分次回输2份供者造血干细胞，回输MNC 1.38×10^8^/kg，CD34^+^细胞0.98×10^6^/kg。未出现明显移植物抗宿主反应（GVHR）。

**图1:**
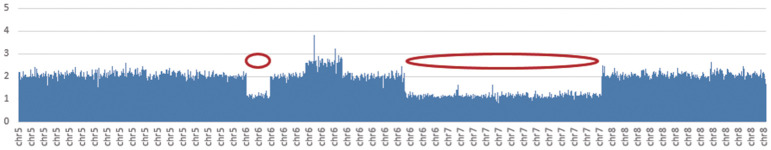
骨髓增生异常综合征患者免疫缺陷基因高通量测序：7号染色体整体缺失；6p25.3p25.2区域（391738～3113573）存在杂合缺失；6q21q25.3区域存在杂合缺失（IRF4、RIPK1）、IFNGR1；其他突变包括STX11、TNFAIP3、TRAF3IP2、ZBTB24、GATA2［c.1081C>G（p.R361G）］、EPG5［c.1572-1G>T（splicing）］、EPG5［c.1963A>G（p.S655G）］、RUNX1、RTEL1、BRCA1、OAS1、IL7、KMT2A、DNMT3B、SLC37A4

**图2:**
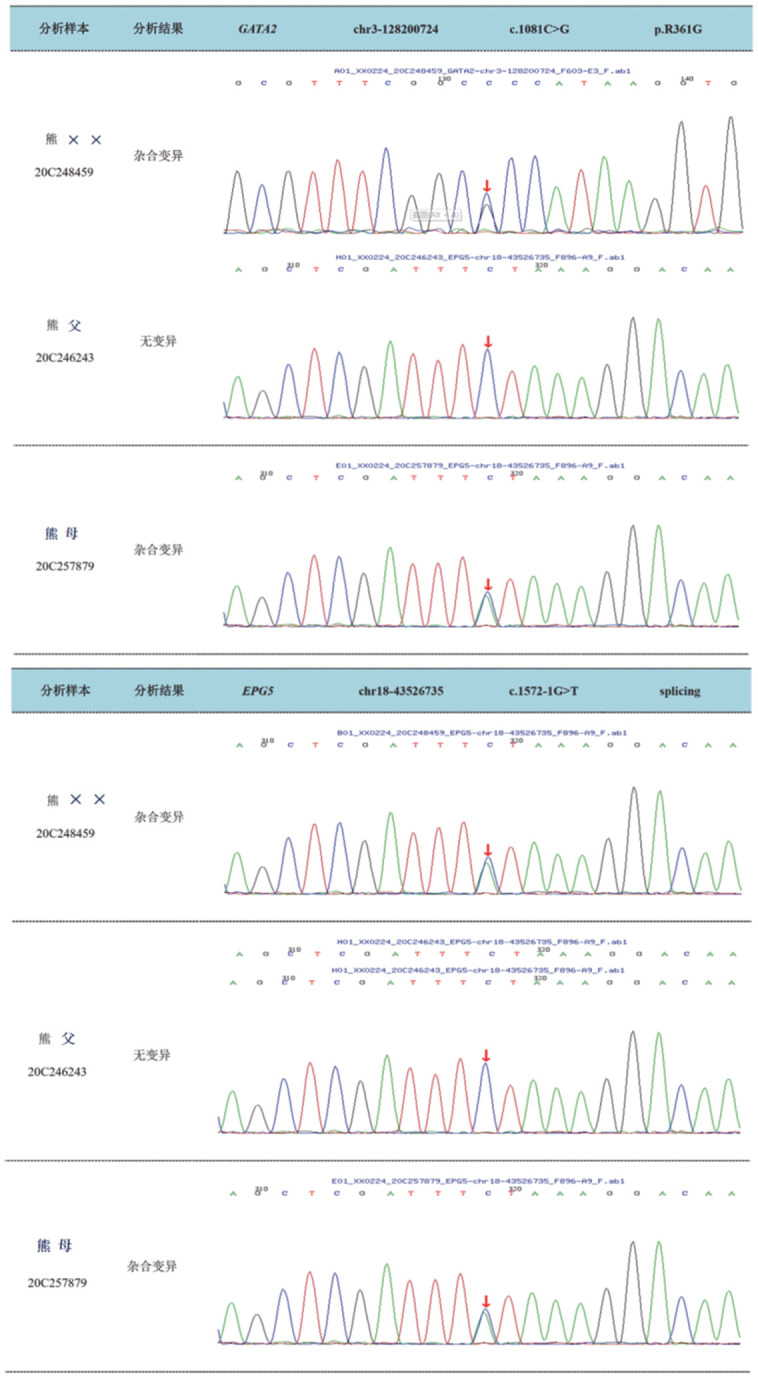
骨髓增生异常综合征患者GATA2及EPG5基因的Sanger家系验证

患者现状：经输注4次供者干细胞后，患者出现的变化为发热频次由每日2～3次变为每日1次，退热所需时间明显缩短，此后体温恢复正常；全身软组织病灶结痂，肛瘘瘘口术后伤口未再渗液，经普外科以及整形美容科诊断为伤口不愈合、伤口机化；仍呈全血细胞减少、输血依赖状态；监测骨髓，期间肿瘤细胞有所控制，但末次复查骨髓，肿瘤细胞比例增至32％，提示进展为急性髓系白血病（AML）。

## 讨论及文献复习

GATA2基因编码造血发育的重要转录因子，通过两个锌指结构（ZF1和ZF2）与上千个基因的GATA结合结构域作用[1]–[3]；其功能主要是以剂量依赖的方式，通过与其他转录因子协同作用，作用于造血祖细胞以调控早期造血。GATA2基因的胚系突变涉及截短突变，多导致ZF2的缺失。此外，目前认为ZF2内的错义突变和位于GATA2-9.5 kb调控区的非编码区变异导致单倍体剂量不足，占比较高的突变类型还有同义突变。GATA2单倍体剂量不足可导致血细胞减少及免疫缺陷，导致携带该突变的患儿在婴儿或儿童期即出现造血、免疫和淋巴系统的症状，并伴有反复感染，进展为MDS/AML的风险高[4]。

GATA2突变相关的异常易向髓系肿瘤转化，但其驱动突变的原因和机制尚不清楚。2016版WHO髓系肿瘤分类提出，相较其他胚系基因改变而言，伴GATA2胚系突变的患者进展为MDS、MDS/骨髓增殖性肿瘤（MPN）以及AML比例最高。GATA2缺陷综合征在成人原发性MDS中总的发生率为7％，在骨髓增生异常综合征伴原始细胞增多（MDS-EB）中发生率达15％；儿童患者中，伴−7和+8异常的MDS患者合并GATA2缺陷发生率高，文献报道，伴−7的MDS患儿中存在37％的GATA2缺陷，+8的MDS患儿中存在16％的GATA2缺陷[5]–[7]。因此，在接诊GATA2合并−7或+8，以及MDS-EB的MDS患者时，应高度警惕合并潜在GATA2缺陷综合征可能。此外，研究发现，GATA2缺陷综合征患者可出现进行性B细胞、单核细胞减少，而监测MDS患者中以上两群细胞是否发生减少可提示GATA2缺陷综合征，此外以上两群细胞显著减少可能是导致患者出现重症感染、以及感染分枝杆菌的原因[8]。治疗方面，目前无统一认识或指南，有学者主张个体化用药调整，也有观点认为化疗对于此类患者不仅获益度有限，且化疗本身可导致严重感染等并发症，故不主张进行化疗，并应尽可能避免免疫抑制剂治疗[9]。异基因造血干细胞移植作为目前唯一的可根治GATA2缺陷综合征的治疗手段，最佳的移植时机是MDS进入低增生时期，GATA2缺陷综合征进入MDS/AL阶段，或者在患者发生严重脏器功能不全前进行[9]–[10]。生存率方面，GATA2免疫缺陷综合征患者总生存率由54％到86％不等[11]。

由于GATA2缺陷综合征的发病中位年龄在17岁左右，有部分患者直至30岁左右才开始出现相关临床表现，故对于GATA2缺陷综合征的诊断、尤其是伴MDS或AML的患者诊断是不及时的[12]–[13]。建议在接诊伴GATA2突变的髓系肿瘤患者时，应注意体细胞检测进行胚系筛查及家系调查，警惕合并GATA2突变相关免疫缺陷及其他综合征的可能性。我们接诊的此例17岁患者，幼时罹患结核；胞弟11岁死于重症水痘，父亲有口腔溃疡发作史，祖母在29岁死于感染，由于弟弟以及祖母已经去世，无法做进一步基因检查验证两位亲属均为GATA2缺陷综合征所致；同时因GATA2缺陷综合征临床表现可以呈隐匿性，发病年龄可以是成年以后[14]，可解释父亲携带该致病基因但目前无明显临床症状。此例患者在诊断为MDS后接受1个疗程去甲基化方案治疗，此后反复发生多发严重感染、进行性全血细胞减少并伴B细胞群以及单核细胞群的显著减少并进展为AML，尽管期间尽可能为其创造移植时机，但各种感染导致移植不能进行，虽尝试输注供者干细胞后感染以及肿瘤细胞有所控制，整体情况未发生逆转，目前未寻找到明显移植时机。

值得一提的是，目前有超过100种不同GATA2胚系基因突变报道，该患者所涉及的GATA2突变位点p.R361G已经研究证实该突变导致GATA2功能缺失DNA结合能力及转化活化能力降低[15]。此例患者同时合并遗传自母亲的EPG5基因突变，EPG5突变可导致Vici综合征，这是一种罕见的常染色体隐性遗传病，涉及全身多个系统的紊乱，主要的临床表现可有联合免疫缺陷[16]。因此，此例患者最终不良结果是否由合并EPG5突变等其他基因多重打击所致目前尚不能得到证实。

通过对该例患者的分析，我们主张：对于伴GATA2突变的MDS，尤其在伴−7和+8异常，MDS-EB患者以及个人史或家族史中有反复感染病史或早期死于感染的患者时，应注意：①GATA2缺陷综合征的筛查；②完善基因测序（如NGS）和家系调查；③尽早行异基因移植治疗；④移植前避免接触化疗及去甲基化药物，避免由此诱发反复、致命的感染及深部脓肿形成等导致移植时机的丧失，错失救治时机。
